# Prevalence of Third-party Tracking on Medical Journal Websites

**DOI:** 10.1001/jamahealthforum.2022.0167

**Published:** 2022-03-18

**Authors:** Ravi Gupta, Ari B. Friedman, Matthew S. McCoy

**Affiliations:** 1 National Clinician Scholars Program at the University of Pennsylvania and Corporal Michael J. Crescenz VA Medical Center, Philadelphia; 2 Division of General Internal Medicine, Department of Medicine, Perelman School of Medicine, University of Pennsylvania, Philadelphia; 3 Leonard Davis Institute of Health Economics, University of Pennsylvania, Philadelphia; 4 Department of Emergency Medicine, Perelman School of Medicine, University of Pennsylvania, Philadelphia; 5 Department of Medical Ethics and Health Policy, Perelman School of Medicine, University of Pennsylvania, Philadelphia

## Abstract

This cross-sectional study investigates web tracking prevalence and characteristics on medical journal websites.

## Introduction

Web tracking tools allow third-party advertisers to compile detailed information about individuals based on their browsing behavior.^1^ Prior research has found that third-party tracking is prevalent on consumer-facing health information websites, eliciting concerns about privacy risks for patients.^1^ Tracking on medical journal websites raises unique ethical and policy considerations because it may help pharmaceutical companies and health care advertisers profile clinicians based on which articles they access. This information can be used to serve clinicians advertising targeted to medical specialties and areas of professional interest inferred from their browsing histories, potentially contributing to undue pharmaceutical industry influence on clinical practice.^2^ Thus, we investigated web tracking prevalence and characteristics on medical journal websites.

## Methods

In this cross-sectional study, we identified all journals with an impact factor of 2.0 or higher in clinically relevant subcategories of the Web of Science’s life sciences and biomedical category.^3^ We visited each journal’s home page using webXray, a tool that detects third-party tracking on websites.^1^ For each journal’s home page, we recorded third-party data requests, which are important because they initiate data transfers from a user’s computer to third parties. We also recorded the presence of third-party cookies, data stored on a user’s computer that frequently serve as persistent identifiers and enable third parties to track individuals across multiple websites.

We calculated the percentage of journals with a third-party data request or cookie and the median number of data requests and cookies per journal home page overall and by journal impact factor. We calculated the most prevalent tracking entities across all web pages. We performed Google searches for the top 5 most prevalent tracking entities’ advertising policies and marketing segment disclosures to determine whether they allowed pharmaceutical advertising and medical profession–specific ad targeting. Data were analyzed in October 2021. This study followed the Strengthening the Reporting of Observational Studies in Epidemiology (STROBE) reporting guidelines for cross-sectional studies. We performed χ^2^ and Kruskal-Wallis tests to examine associations between journal third-party data requests or cookies and impact factor. All statistical tests were 2-tailed and used a *P* value of .05 as a threshold for significance. Analyses were conducted in Stata, version 16 (StataCorp LLC).

## Results

Overall, 1599 of 1605 (99%; 95% CI, 99%-100%) medical journal home pages included a third-party data request, and 1239 (77%; 95% CI, 75%-79%) included a third-party cookie, without significant differences by impact factor (Table). Journal home pages had a median (IQR) of 8 (1-17) third-party cookies with no differences by impact factor. The median (IQR) number of third-party data requests per journal was 31 (11-45), with higher-impact journals significantly associated with fewer third-party requests (Table).

**Table.** Prevalence of Third-party Tracking on Medical Journal Websites

Characteristic	Overall	Impact factor				*P* value
		1-5	5.01-10	10.01-15	15.01-24	
No. (%) of websites	1605 (100)	1278 (80)	236 (15)	40 (2)	51 (3)	<.001^a^
Websites, No. (%) [95% CI]						
With a third-party data request	1599 (>99) [99-100]	1273 (>99) [99-100]	236 (100) [98-100]	39 (98) [87-100]	51 (100) [93-100]	.11^b^
With a third-party cookie	1239 (77) [75-79]	978 (77) [74-79]	192 (81) [76-86]	32 (80) [67-93]	37 (73) [60-85]	.33^b^
Third-party cookies per journal home page, median (IQR)	8 (1-17)	8 (1-20)	8 (1.5-14)	7.5 (1-18)	8 (0-14)	.95^c^
Third-party requests per journal home page, median (IQR)	31 (11-45)	33 (9-47)	26.5 (16-42)	20 (13-40)	19 (5-33)	.002^c^

^a^ χ^2^ goodness-of-fit.

^b^ Pearson χ^2^.

^c^ Kruskal-Wallis.

Nearly all (1593 of 1605 [99%]) journal home pages included a data request from a third-party entity owned by Alphabet, Google’s parent company. Data requests from entities owned by Twitter, Facebook, Oracle, and Adobe occurred on at least 40% of journal home pages (Figure). Marketing segment disclosures were found only for Oracle^4^ and Adobe Inc,^5^ both of which allow medical profession–specific ad targeting. However, all 5 top tracking entities allow pharmaceutical advertising.

**Figure.**
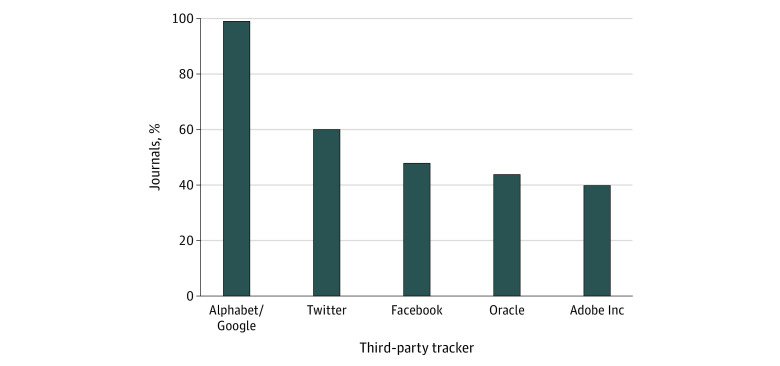
Most Prevalent Third-party Tracking Entities on Medical Journal Websites The y-axis represents the percentage of medical journal websites with a third-party data request from a given tracking entity.

## Discussion

This cross-sectional study demonstrated that 99% of medical journals with an impact factor of 2.0 or higher expose visitors to third-party tracking by entities that work with pharmaceutical advertisers. Although similar levels of tracking have been found in health-related websites, tracking on journal websites raises distinctive policy concerns because it may facilitate targeted advertising to clinicians. While targeted advertising can increase knowledge of new therapeutics, it can also sway clinicians’ prescribing patterns toward therapies with limited evidence of efficacy and cost-effectiveness.^6^

This study had limitations. Results may not be generalizable to medical journals with an impact factor of 2.0 or lower. Additionally, marketing segment disclosures for 3 of 5 top tracking entities could not be located, and those that were located may be out of date. Finally, this study did not assess how accessing articles through library proxies may alter tracking.

Given growing concerns over digital health privacy risks and pharmaceutical advertising to clinicians, medical journal editors and publishers should monitor and assess the potential impact of third-party tracking on journal websites. Further research is needed to determine how tracking information influences targeted advertising to clinicians.
